# Academy of Stomatology

**Published:** 1900-05

**Authors:** 

**Affiliations:** Academy of Stomatology


### ACADEMY OF STOMATOLOGY.

A regular monthly meeting of the Academy of Stomatology was held at the rooms of the Academy, 1731 Chestnut Street, Philadelphia, on Tuesday, December 26, 1899, the President, Dr. E. C. Kirk, in the chair.

A paper entitled "Common-Sense Occlusion, or 'Bite,'" was read by Dr. W. Warrington Evans, of Washington, D. C.

(For Dr. Evans's paper, see page 221.)

### DISCUSSION.

*The President.*—This is a very interesting and practical subject. I have no doubt that you are all anxious to break a lance with the essayist, and the subject is now open for your discussion. I see a number of gentlemen present who are not members of the society, and I wish to announce that the courtesy of the floor is always extended to visitors. We shall be glad to hear from our visiting friends. I shall ask Dr. Guilford to open the discussion.

*Dr. S. H. Guilford.*—I would like to ask Dr. Evans whether he thinks he gains much by having the mass of gutta-percha worked up in that way, instead of having the wax on the thin gutta-percha? I understand that he uses the gutta-percha for the purpose of giving greater stability, and on some of the trial-plates it is placed around it a quarter of an inch. He said it was desirable to have this plate as nearly like the finished plate as possible.

*Dr. W. Warrington Evans.*—Yes. Those two plates, or models, that were passed around were simply pressed up hastily last night for this demonstration, and are thicker than I usually have them, but you will notice that the other plates which have the pieces of wax attached are thinner and have been better carved to shape. There is some thickness in the centre of the palate, which is not so noticeable to the patient, provided you have the bulkiness on the outside of the process removed. It would be, of course, greatly different in the finished plate, as one cannot cut the gutta-percha down so closely as in the finished plate. The point is well taken and requires explanation. Of course, when I said the "finished plate," I had reference more to the external than to the internal surface. I merely approximate the form. I would not make the palatal portion of my base-plate as thin as I would make a finished vulcanite plate for the mouth, as it needs stability. It is simply used to take the bite, and is not used after articulating the model. When "waxing up" the teeth I use nothing thicker than the thinnest rolled pink paraffin and wax, as it comes from the depots. Sometimes I use wax as thin as that from which artists make artificial flowers. I do not have two trial-plates, but use one base-plate to get the bite with, the other to set the teeth on after getting the bite. Neither are trial-plates, because I never see my patient until I finish the work. I never try the teeth in the mouth on the plate.

*Dr. Guilford.*—What advantage is there? There is a great deal more difficulty in working out that mass of gutta-percha to represent the outline of the teeth than to have the gutta-percha merely laid over the ridge and build it up with wax. Does it not require more time?

*Dr. Evans.*—It requires more time, and it is harder to do, without doubt, but when it is done it is rigid in form and remains firm, which is particularly advantageous in the summer time, for if one is going to spend ten or fifteen minutes with a patient getting a correct expression of the mouth, one will find that the ordinary wax will change its shape in the mouth owing to lack of rigidity. That has been my experience, to say nothing of time lost by trying the teeth mounted on wax in the mouth.

*Dr. William H. Trueman.*—I think the present is a very good time for reducing the rude methods of the past to a more scientific basis and getting down to finer lines. During the reading of the paper my mind has referred to the old-time methods of taking articulations, when a mass of wax was made into a ball and placed between the two plates while they were in the mouth (gold and silver were generally used), the patient directed to close, and when in the operator's judgment the jaws were closed enough he took it out of the mouth and sent it to the laboratory; the mechanical dentist did the rest. It is astonishing how accurate they frequently were. It was a very rude way, but the operators at that time were used to taking articulations by that method, and knew about how to do it; and as spiral springs were generally used, a little malarticulation was less important than it is now. If we would try the same methods now, we would fail probably every time, but they did it and succeeded very frequently. Then, after that, the wax was divided, a portion was placed on each plate, and very accurately curved to the contour line of the intended teeth. That was sent to the laboratory, and the workman was expected to make the teeth exactly conform to these wax moulds. Indeed, in some cases we were required to make a plaster mould, and to fit the teeth up to that. Wherever that was done it was a failure, for the reason that the porcelain teeth could not be made to conform to the outline of the wax. It was easy to carve the wax, but difficult to go to the dental depot and find teeth that would fit. When the hand-curved blocks were used, those wax-guides came into use. In my practice, I begin to take the articulation when I first see the patient. In conversing with them, I try to fix in my mind what seems to be the normal and natural expression of the individual, and, having that in my mind when the time comes, I try to catch the point when that expression has been reached. I find it is a very good idea. I do not depend so much on carving the wax as I do on getting the idea in my mind of the natural expression. I think it is very good to reduce these things down, so far as may be done, to mathematical lines for the benefit of those who are beginning; it fixes in their minds what is a normal denture, and assists them very materially in arriving at that which is right and proper. At the same time, you must always remember that there is an eternal fitness of things to be considered. Teeth may be made according to mathematical lines and yet when placed in the mouth may be a complete botch, because they do not harmonize with that which the individual requires.

There is another point we very frequently lose sight of when speaking of the natural outline and the natural teeth in this connection. We forget that we never place an artificial denture in the same mouth from which the teeth have been extracted. When the time comes for placing an artificial denture in, that mouth has changed so much that it is not the same mouth. It is utterly impossible to restore the expression as it was when the natural teeth were there. If you consider the difference between the gum when the teeth have been just extracted and the gum when shrinkage has taken place and got down to what we sometimes call "bed-rock," you will see that it is utterly impossible to restore the features as they once were. It is utterly impossible to put back the natural teeth after that has taken place. There is a general contraction of the tissues, and the best that we can do is to study the features and make them harmonize; make them look well, in other words. I think this is not always considered. How frequently I see remarks made in journals by speakers to the effect that the teeth at the depots are too small, that they are not the natural shape. The molars are particularly complained of as being too narrow, and as not having sufficient masticating surface, and the fact that there is not as much room at our disposal as there was when the normal denture was there is lost sight of. There has been a general contraction of the tissues, and the mouth is smaller. There is a great difference between the shrunken tissues and these same tissues when the natural teeth were there.

In reference to these expedients for making trial-plates, I much prefer swaged-up plate made of block tin. Where we have conveniences in the laboratory for making them they can be gotten up almost as quickly as these expedients can be. The tin plates are made to the approximate thickness of the intended vulcanite, and I find them very much more satisfactory in adjusting to the mouth, in getting the articulation, and adjusting the teeth when they are waxed in place. My own experience with wax plates and other devices has been that I generally have had to do my work over a second time. That is, probably, because I am not accustomed to using plates of that kind, so I find it more satisfactory to make block-tin plates. In all these matters that is best to which the operator is most accustomed,—that is, more accustomed to using. Some of my friends use wax plates and have no trouble. I prefer the block tin.

I was very much interested and impressed a year or two ago, upon visiting a dentist in the interior of Pennsylvania, to find him using Dr. Bonwill's articulator. I asked him if he used it to play with. He said he used it to work with—very emphatically. I said, "Do you find it useful?" He said, "Why, I cannot do without it." He never met Dr. Bonwill, nor did he know Dr. Bonwill's ideas as regards the articulation and articulator, and yet had, in studying out the articulator, struck upon the idea. He said he found it very useful, indeed. Very frequently patients came to him from a distance, and he took the impression—took the articulation—and selected a set of teeth; the patients went away twenty or thirty miles, and when the case was finished he sent them by mail, and he very rarely had any changes to make. He had later seen the cases sent out that way when patients in passing through the town called on him, and he found them articulating very nicely, indeed. He said that until he had learned to use that articulator he was not able to work so accurately. I have tried to use it; several times Dr. Bonwill has tried to explain to me how to use it, but my head was too thick to take it in. I have not been able to use it with any degree of satisfaction, and yet those who seem to have done so find it practically useful.

I think one of Dr. Bonwill's great points was to so arrange the teeth that, no matter in what position the jaws might come together, there were always three points of contact,—one in the front and one on each side,—and so it was impossible in closing the jaws to tilt the plates. He explained to me a number of times how that could be done, how he accomplished it, but I have not been able to do it in my practice.

In arranging these matters I try to study the case and arrange it as experience seems to dictate; at the same time I think it is a very good idea to have these various practical points worked down to fine lines. We have learned by experience, while those who come after us will learn more quickly, and probably better, by precept.

*Dr. McCullum.*—I have been using Dr. Bonwill's articulator for three or four years, and like it for articulating teeth by Dr. Bonwill's system, which appeals to me because it is a system, and the only one taught. I never learned to set up teeth systematically until I got Dr. Bonwill's system of articulation. I finished a set of teeth to-day articulated by this method, and I found the occlusion very good.

*Dr. M. H. Cryer.*—I would like to say a few words in regard to this equilateral triangle of the lower jaw. When Dr. Bonwill made a visit to the University, about a year ago, we measured nearly all the lower jaws in the Dental Museum, and did not find a single jaw that came up to the requirements of the equilateral triangle as claimed by him. They vary as much as an inch one way or the other; the condyles may be fully one inch closer than the distance from the centre of the condyle to the inner corner of the cutting edge of the central incisor, or again they may be one inch under. Yesterday while at the Dental Department of the University of Pennsylvania I picked up a skull, and taking a pair of dividers, measured the lower jaw, and found that the measurements between the centres of the condyles and from the centre of the condyle to the central incisors were exactly the same, giving a true equilateral triangle. Upon further investigation I found this skull had belonged to the collection of the late Dr. Bonwill, which his daughter had presented to the Department of Dentistry of the University of Pennsylvania. I presume that it was upon measurements from this skull that Dr. Bonwill based his discovery. Had he examined a skull of one of the Fan tribe of Africa he would have found the distance from the condyles to the incisors to have been three-fourths of an inch longer to the centre of the other condyle; or, had he taken the skull of a Chinese or a North American Indian, he would have found the reverse condition. These skulls are in the Department of Dentistry of the University of Pennsylvania; those who may visit the museum can make the measurements for themselves. There are examples from many races, among them Europeans, Asiatics, Africans, and various tribes of the North American Indians, and the only skull in that collection that will give you the equilateral triangle is the one belonging to Dr. Bonwill's collection. This gives one more illustration of the fact, so hard to understand, that the typical jaw, or the true type of any physical perfection, is the one most difficult to obtain, and, as our text-books generally only define these, we seem to be always dealing with abnormalities.

*Dr. Albert P. Brubaker.*—There is but one word I would like to say in reference to the data and the measurements of the equilateral triangle. If I recollect aright, Dr. Bonwill stated in his original paper that he had examined the jaws of some two thousand skulls in the collection of the Academy of Natural Sciences in this city, which embraces skulls of a great variety of races, and that his results represented the general average of many measurements.

I would also call attention to the fact that the want of correspondence between the scientific interpretation of the equilateral triangle and the practical application of the same is largely due to an arbitrary and non-geometrical construction not only of the relative sizes of the teeth, but also of their relative positions. This method of Dr. Bonwill has been carefully analyzed and refuted by Dr. Alfred Gysi, of Zurich. Those who have carefully read Dr. Gysi's paper will be readily convinced that Dr. Bonwill's construction is geometrically and physiologically incorrect.

*Dr. G. C. Chance.*—I am very much in favor of this old-fashioned articulator that Dr. Evans has suggested. I have seen it work beautifully where "all others failed."

*Dr. H. Gaskill.*—Dr. Bonwill one evening said that he had never seen but one case in which the natural teeth had movement contacts corresponding to those given to artificial teeth by his method of articulation. That was in the case of an elderly gentleman who had one or two teeth missing, but in the movement of the jaw there were three points of contact of the teeth.

*Dr. A. H. Thompson.*—The essayist did not touch upon the question of lateral movement in occlusion, which, of course, is a very important one in articulating the teeth, and while the main position is that in which the jaw is at rest, still we must consider the movements of the jaw in the process of mastication of food, which is often a very important matter. There is very considerable excursion of the human jaws allowed, but owing to the depth of the glenoid cavity in the human subject it is somewhat restricted. We are in the habit of giving the patient some directions regarding the use of artificial plates, to bite just so, or to use them in particular ways. I think that Dr. Bonwill has really given us some ideas as to the movements of occlusion which are valuable and which may be applied to the adjustment of artificial teeth with reference to mastication.

*Dr. James Truman.*—I was very much gratified with Dr. Evans's exposition of his subject, all the more, perhaps, because I always used, when I was making artificial teeth, the old plaster extension articulator, and I can fully endorse its value. I do not think that I have at any time articulated a set with the modern articulator, and have practically used the method of Dr. Evans, although not making use of gutta-percha. I have never felt that confidence in articulation which would enable me to send a set of teeth by mail with the anticipation that it would answer without subsequent grinding. I hardly can understand how that can be accomplished. I would like to hear from Dr. Evans on that point.

*Dr. G. W. Cupit.*—One point has been brought out very nicely, and that is, that in the arrangement of the bites the outline of the finished plates is followed more closely. Dr. Evans works more accurately than is the custom of the average dentist. We are apt to make our bites thicker and fuller, possibly interfering with the proper occlusion and proper expression, and for that reason we do not get accuracy of occlusion or expression. I think the accuracy in the finished plate is due to the accuracy of the bite-plates. If they fit nicely and are well shaped, so as not to interfere with the action of the muscles or the expression of the lips, we may naturally expect the finished plate to be the same, and to me this is where the accuracy is accomplished, and is why the finished plates can be sent by mail or express with comparative certainty that they will be correct.

At the same time, I wish to discourage the practice of taking what is called the "mush bite," putting in a large lump of wax, sufficient to nearly fill the mouth, and having the jaw come into contact with that, and feeling that it is a correct or even an approximate bite. It is not. I think the more nearly our bites and bite-plates are arranged to represent the finished plates the more nearly correct will be the finished plate.

*Dr. S. H. Guilford.*—We all know what Dr. Bonwill's skill was, and we know of Dr. Evans's skill. Dr. Bonwill insisted upon using his anatomical articulator, and in that articulator he not only got the vertical, but also the lateral and rotary motions, and he also, in addition, by means of a skeleton articulator, was enabled to look from the inside and see how the inner cusps occluded. He thought it was essential, and that a set of teeth could not be properly arranged except by an articulator made on that principle. Dr. Evans says he has no need of an articulator of that kind, but uses a fixed articulator, yet he meets with the same success that Dr. Bonwill did. Therefore it seems to resolve itself largely into a matter of personal equation, a great deal depending on the individual, each doing his work in his own way. Dr. Evans works with exceeding nicety and exactness, as he has shown to-night, but, aside from that, I do not see that there is anything of great importance in his methods; in other words, two men, equally skilful, get the same results by different methods. So it all depends very largely on getting correct occlusion in the first place, no matter how you get it, and after that is done, setting the teeth in such a manner that they will restore the expression and properly perform the functions of mastication and speech.

*Dr. Evans.*—Gentlemen, you certainly have been very kind in your criticisms. I once asked a gentleman, who is very skilled as a government photographer, what plate was the best that I could use for photographing. He said, "There are several good plates, but my advice is to select one of those plates and master it. If you change from plate to plate, you will not succeed, but if you will take one good make of plate and learn all its difficulties, you will be able to manage photography." And so it is in regard to dentistry. Now, as to taking the bite. There may be some who will accomplish it by using wax alone, others with gutta-percha or tin, and so on; but, after all, the secret of mailing a product which will not require grinding does not depend upon my articulator or occlusion-plate alone, by any means. If after getting perfect occlusion I do not give attention to all the little details of manipulation of materials, they would not be so exact as not to require articulation in the mouth. In vulcanite work, if you pack very heavily or have a very thick plate, where you can have contraction or expansion, or place a large amount of material in between the two halves of the flask and attempt to force it down with bolts, you will have unsatisfactory results, and no matter how perfect your occlusion may have been in the beginning, you will have grinding to do. On the other hand, learn to pack extremely close with vulcanite, so that there is a very thin margin of excess, and use a flask without bolts, such as have come in lately, with a spring clamp to close it. If there happen to be the least inaccuracy in closing the flask, the spring clamp will complete the operation when the material has become more plastic under the high heat of vulcanizing. While these are small details, yet they are essential to obtain perfect results; you must follow out all details.

In reply to Dr. Cryer's remarks relative to measurements of the jaw, I may say that my experience happens to be different from his. I believe a great deal depends upon the nationality of the skull examined. He happened to have a line of skulls from certain nationalities at the University of Pennsylvania. I happened to go to the Government Museum, where there are two or three thousand skulls of Indians and about fifteen hundred or so of other skulls,—Caucasians, negroes, and so on,—and my observation was that more than one-half of the lower jaws would stand the measurement for an equilateral triangle. The other half would vary not more than one-quarter of an inch from it. As I said before, it does not make any difference whether it is an equilateral triangle or a trapezoid, provided we happen to have a means of taking the individual bite. We must adapt ourselves to circumstances. I thank you very much, gentlemen, for your kind attention.

Adjourned.

OTTO E. INGLIS,
*Editor Academy of Stomatology.*

